# Supplementary material for: Effects of vitamin D on inflammatory and oxidative stress responses of human bronchial epithelial cells exposed to particulate matter
Source: PLoS One. 2018 Aug 29;13(8):e0200040. doi: 10.1371/journal.pone.0200040 (PMC6114286; doi:10.1371/journal.pone.0200040)
Supplement: S1 Table — (PDF) [file pone.0200040.s001.pdf]

S1 Table: Taqman primer probesets

Gene	Fluorescent Label	Primer – Probeset ID
18S	VIC	Hs9999990_s1
CYP24A1	FAM	Hs00167999_m1
CYP27B1	FAM	Hs00168017_m1
CAMP	FAM	Hs001891038_m1
CSF2	FAM	Hs00929873_m1
CXCL10	FAM	Hs01124251_g1
G6PD	FAM	Hs00166169_m1
IL6	FAM	Hs00985639_m1
IL8	FAM	Hs00174103_m1
IL24	FAM	Hs01114274_m1
TGFB2	FAM	Hs00234244_m1
VDR	FAM	Hs01045846_m1
