# Supplementary material for: Effects of vitamin D on inflammatory and oxidative stress responses of human bronchial epithelial cells exposed to particulate matter
Source: PLoS One. 2018 Aug 29;13(8):e0200040. doi: 10.1371/journal.pone.0200040 (PMC6114286; doi:10.1371/journal.pone.0200040)
Supplement: S2 Table — Gene expression fold-changes in a transcription microarray of HBECs cultured for 24 hours with/without 50μg/ml NIST stimulation in the presence/absence of 100nM 1,25(OH)2D3. Short-list of genes showing both differential expression with NIST stimulation (fold change ≥ ± 1.4) and differential expression with vitamin D treatment in presence of NIST stimulation (fold change ≥ ± 1.4). (PDF) [file pone.0200040.s002.pdf]

S2 Table: Genes showing differential regulation by both NIST stimulation and vitamin D

Gene ID	p value	Fold-Change	Direction Control vs. 1,25(OH)$_2$D3	p value	Fold-Change	Direction Control vs. NIST	p value	Fold-Change	Direction NIST vs. NIST+1,25(OH)$_2$D3
IL1RL1	0.0465043	-2.10531	Control down vs 1,25(OH)2D3	0.168072	-1.62235	Control down vs NIST	0.011141	-2.79324	NIST down vs NIST+1,25(OH)2D3
G0S2	0.00914142	-1.90065	Control down vs 1,25(OH)2D3	0.00309638	-2.1762	Control down vs NIST	0.000624146	-2.70591	NIST down vs NIST+1,25(OH)2D3
KLK6	0.00179259	-1.86993	Control down vs 1,25(OH)2D3	0.0245908	-1.47087	Control down vs NIST	0.000123887	-2.50339	NIST down vs NIST+1,25(OH)2D3
TGFB2	0.0270176	-1.61114	Control down vs 1,25(OH)2D3	0.00894347	1.82264	Control up vs NIST	0.00282454	-2.08522	NIST down vs NIST+1,25(OH)2D3
CLDN7	0.298211	-1.37284	Control down vs 1,25(OH)2D3	0.0601412	-1.85293	Control down vs NIST	0.0678588	-1.81381	NIST down vs NIST+1,25(OH)2D3
SERPINB1	0.0084738	-1.64167	Control down vs 1,25(OH)2D3	0.000730376	-2.09662	Control down vs NIST	0.00330487	-1.79556	NIST down vs NIST+1,25(OH)2D3
CLCF1	0.0804403	-1.29685	Control down vs 1,25(OH)2D3	0.0176374	-1.4661	Control down vs NIST	0.00192102	-1.76969	NIST down vs NIST+1,25(OH)2D3
TINAGL1	0.0560214	-1.53893	Control down vs 1,25(OH)2D3	0.0528185	-1.54989	Control down vs NIST	0.0204494	-1.73685	NIST down vs NIST+1,25(OH)2D3
CALML3	0.0473789	-1.55187	Control down vs 1,25(OH)2D3	0.00746459	1.92993	Control up vs NIST	0.0193797	-1.72279	NIST down vs NIST+1,25(OH)2D3
COL1A1	0.00843668	-1.67318	Control down vs 1,25(OH)2D3	0.0181665	1.55538	Control up vs NIST	0.00978423	-1.6495	NIST down vs NIST+1,25(OH)2D3
FNDC4	0.0171438	-1.36635	Control down vs 1,25(OH)2D3	0.00562058	-1.47242	Control down vs NIST	0.00116336	-1.64913	NIST down vs NIST+1,25(OH)2D3
TMEM132A	0.078646	-1.54403	Control down vs 1,25(OH)2D3	0.0520706	-1.63239	Control down vs NIST	0.0501363	-1.6407	NIST down vs NIST+1,25(OH)2D3
ZNF185	0.00548689	-1.56017	Control down vs 1,25(OH)2D3	0.0200145	-1.41295	Control down vs NIST	0.00568852	-1.55575	NIST down vs NIST+1,25(OH)2D3
LOC644774	0.23192	-1.54818	Control down vs 1,25(OH)2D3	0.201853	-1.5991	Control down vs NIST	0.240484	-1.53497	NIST down vs NIST+1,25(OH)2D3
HSD17B2	0.0212805	-1.3591	Control down vs 1,25(OH)2D3	5.60E-06	-2.84148	Control down vs NIST	0.00370075	-1.53464	NIST down vs NIST+1,25(OH)2D3

CYBA	0.228216	-1.37571	Control down vs 1,25(OH)2D3	0.20045	-1.40608	Control down vs NIST	0.126281	-1.51538	NIST down vs NIST+1,25(OH)2D3
LOC732007	0.297057	-1.40272	Control down vs 1,25(OH)2D3	0.21826	-1.49883	Control down vs NIST	0.218839	-1.498	NIST down vs NIST+1,25(OH)2D3
LHFPL2	0.156393	-1.22409	Control down vs 1,25(OH)2D3	0.0188548	-1.45298	Control down vs NIST	0.0145626	-1.48348	NIST down vs NIST+1,25(OH)2D3
TNFRSF12A	0.283917	-1.52121	Control down vs 1,25(OH)2D3	0.096998	-1.97767	Control down vs NIST	0.319492	-1.4739	NIST down vs NIST+1,25(OH)2D3
FUT3	0.268488	-1.1781	Control down vs 1,25(OH)2D3	0.0120253	-1.54603	Control down vs NIST	0.0234616	-1.46016	NIST down vs NIST+1,25(OH)2D3
LOC653888	0.22172	-1.49047	Control down vs 1,25(OH)2D3	0.167983	-1.57748	Control down vs NIST	0.260954	-1.43999	NIST down vs NIST+1,25(OH)2D3
SLPI	0.29415	-1.26871	Control down vs 1,25(OH)2D3	0.031599	-1.72139	Control down vs NIST	0.125053	-1.43523	NIST down vs NIST+1,25(OH)2D3
PGAM1	0.299795	-1.38669	Control down vs 1,25(OH)2D3	0.276903	-1.41045	Control down vs NIST	0.256957	-1.43284	NIST down vs NIST+1,25(OH)2D3
SEMA6A	0.167059	-1.22372	Control down vs 1,25(OH)2D3	0.0124078	1.51964	Control up vs NIST	0.0258794	-1.43019	NIST down vs NIST+1,25(OH)2D3
PTAFR	0.244684	-1.2013	Control down vs 1,25(OH)2D3	0.028395	-1.46842	Control down vs NIST	0.0406266	-1.42192	NIST down vs NIST+1,25(OH)2D3
MMP9	0.893793	1.03243	Control up vs 1,25(OH)2D3	0.0288699	-1.82859	Control down vs NIST	0.171049	-1.41294	NIST down vs NIST+1,25(OH)2D3
SPRR2G	0.62754	1.09394	Control up vs 1,25(OH)2D3	0.00640393	-1.87983	Control down vs NIST	0.0881717	1.40746	NIST up vs NIST+1,25(OH)2D3
SPRR2C	0.264261	1.1972	Control up vs 1,25(OH)2D3	0.0010141	-2.05692	Control down vs NIST	0.048364	1.41205	NIST up vs NIST+1,25(OH)2D3
MX2	0.62258	1.21054	Control up vs 1,25(OH)2D3	0.142998	-1.82519	Control down vs NIST	0.365557	1.42939	NIST up vs NIST+1,25(OH)2D3
SPRR2F	0.00102203	1.57848	Control up vs 1,25(OH)2D3	2.24E-06	-2.75387	Control down vs NIST	0.00433959	1.4365	NIST up vs NIST+1,25(OH)2D3
SNORA79	0.388657	1.36502	Control up vs 1,25(OH)2D3	0.161591	1.6886	Control up vs NIST	0.318605	1.43718	NIST up vs NIST+1,25(OH)2D3
TOP2A	0.352653	1.12965	Control up vs 1,25(OH)2D3	0.0110559	1.48653	Control up vs NIST	0.016798	1.43946	NIST up vs NIST+1,25(OH)2D3
IFIT2	0.890007	-1.05781	Control down vs 1,25(OH)2D3	0.218496	-1.68637	Control down vs NIST	0.376849	1.4437	NIST up vs NIST+1,25(OH)2D3
LOC100132 240	0.333111	1.29278	Control up vs 1,25(OH)2D3	0.158863	1.47065	Control up vs NIST	0.175731	1.44627	NIST up vs NIST+1,25(OH)2D3

S100A7A	0.69075	1.09231	Control up vs 1,25(OH)2D3	0.0021643	-2.48868	Control down vs NIST	0.109224	1.4651	NIST up vs NIST+1,25(OH)2D3
CDC45L	0.172259	1.28224	Control up vs 1,25(OH)2D3	0.0716714	1.40797	Control up vs NIST	0.0453846	1.47577	NIST up vs NIST+1,25(OH)2D3
SPRR2E	0.246472	1.24643	Control up vs 1,25(OH)2D3	4.83E-05	-3.62496	Control down vs NIST	0.0529816	1.48542	NIST up vs NIST+1,25(OH)2D3
IFI6	0.805071	1.09868	Control up vs 1,25(OH)2D3	0.223558	-1.62254	Control down vs NIST	0.305634	1.49506	NIST up vs NIST+1,25(OH)2D3
SLC7A11	0.419671	1.16406	Control up vs 1,25(OH)2D3	0.000213763	-2.9153	Control down vs NIST	0.0510411	1.49799	NIST up vs NIST+1,25(OH)2D3
IL6	0.049738	1.42547	Control up vs 1,25(OH)2D3	0.00121734	-2.06736	Control down vs NIST	0.0279799	1.50594	NIST up vs NIST+1,25(OH)2D3
IFIT1	0.989147	1.00702	Control up vs 1,25(OH)2D3	0.146043	-2.21568	Control down vs NIST	0.42431	1.5196	NIST up vs NIST+1,25(OH)2D3
PBK	0.314518	1.16635	Control up vs 1,25(OH)2D3	0.0238827	1.4798	Control up vs NIST	0.0154321	1.53816	NIST up vs NIST+1,25(OH)2D3
IFI44	0.53743	1.24944	Control up vs 1,25(OH)2D3	0.162021	-1.69744	Control down vs NIST	0.231968	1.56083	NIST up vs NIST+1,25(OH)2D3
KIAA1199	0.0166052	1.68302	Control up vs 1,25(OH)2D3	0.085661	1.40815	Control up vs NIST	0.0306761	1.57463	NIST up vs NIST+1,25(OH)2D3
SPRR1A	0.138988	1.21832	Control up vs 1,25(OH)2D3	0.000261189	-2.02378	Control down vs NIST	0.00439874	1.58243	NIST up vs NIST+1,25(OH)2D3
IL24	0.0394541	1.31655	Control up vs 1,25(OH)2D3	3.79E-06	-3.11063	Control down vs NIST	0.00117518	1.7043	NIST up vs NIST+1,25(OH)2D3
IFI44L	0.632328	1.2925	Control up vs 1,25(OH)2D3	0.240531	-1.91763	Control down vs NIST	0.297626	1.7732	NIST up vs NIST+1,25(OH)2D3
RASD1	0.0615367	1.4049	Control up vs 1,25(OH)2D3	0.0115027	-1.65471	Control down vs NIST	0.00211878	1.96993	NIST up vs NIST+1,25(OH)2D3
CXCL10	0.769044	1.12621	Control up vs 1,25(OH)2D3	0.0650825	-2.28168	Control down vs NIST	0.112008	1.99732	NIST up vs NIST+1,25(OH)2D3

Gene expression fold-changes in a transcription microarray of HBECs cultured for 24 hours with/without 50μg/ml NIST stimulation in the presence/absence of 100nM 1,25$(OH)_2$D3. Short-list of genes showing both differential expression with NIST stimulation (fold change ≥ ± 1.4) and differential expression with vitamin D treatment in presence of NIST stimulation (fold change ≥ ± 1.4).
