# Supplementary figures and images for: Effects of vitamin D on inflammatory and oxidative stress responses of human bronchial epithelial cells exposed to particulate matter
Source: PLoS One. 2018 Aug 29;13(8):e0200040. doi: 10.1371/journal.pone.0200040 (PMC6114286; doi:10.1371/journal.pone.0200040)

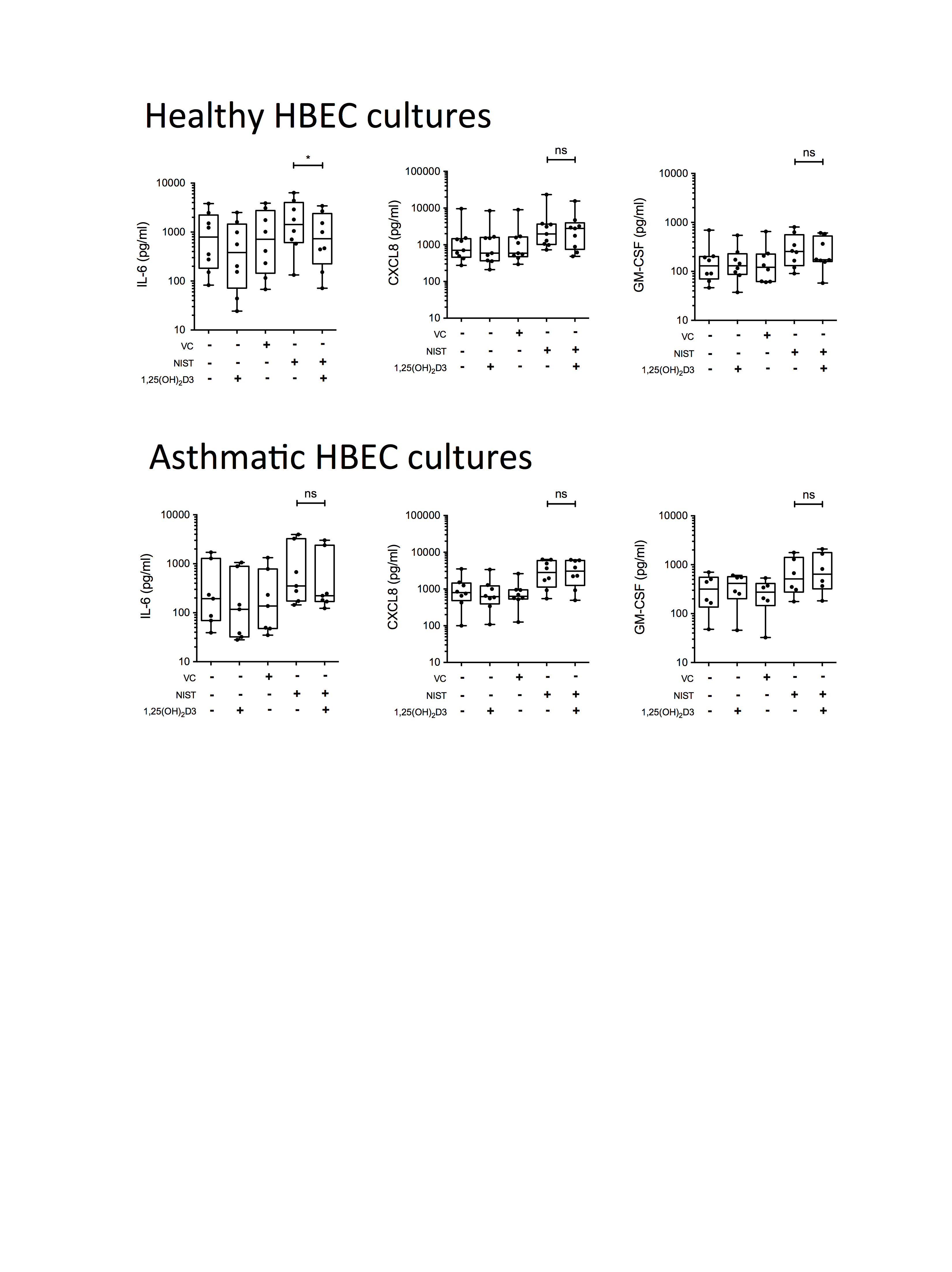

Supplement: S1 Fig — Primary HBECs stimulated for 24 hours with 50μg/ml NIST in the presence/absence of 100nM 1,25(OH)2D3. VC; vehicle control for NIST. Healthy donor cultures: n = 8 for IL-6, n = 9 for CXCL8, n = 8 for GM-CSF. Asthmatic donor cultures: n = 7 for IL-6, n = 8 for CXCL8, n = 6 for GM-CSF. Friedman’s tests with Dunn’s multiple-comparisons tests. Statistical significance: *, p ≤ 0.05. (TIFF) [file pone.0200040.s003.tiff]

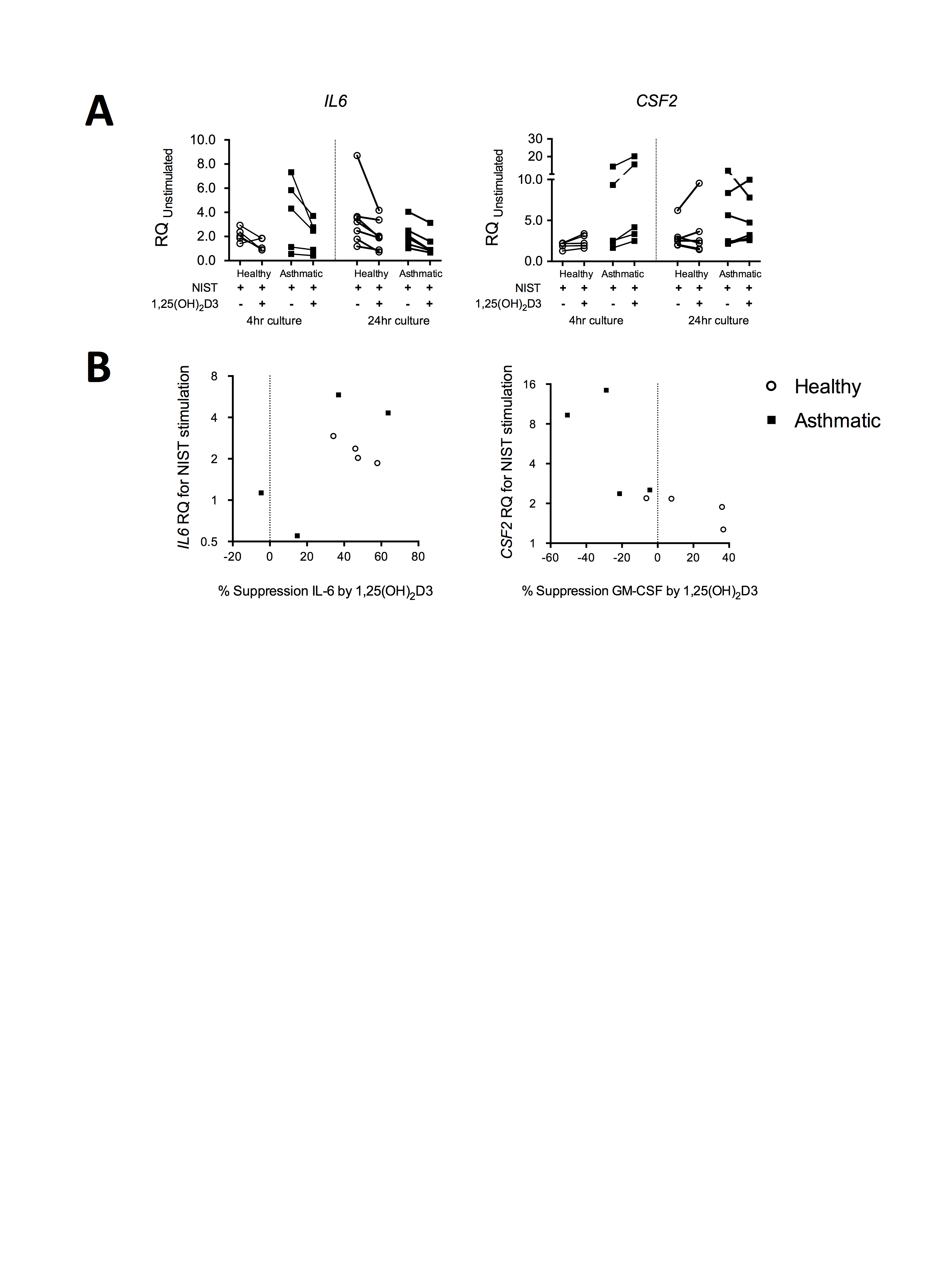

Supplement: S2 Fig — (A) Induction of IL6 and CSF2 by 50μg/ml NIST PM stimulation in the presence / absence of 100nM 1,25(OH)2D3 in 4 hour and 24 hour cultures of HBECs from healthy and asthmatic donors. H, Healthy donor HBECs; A, Asthmatic donor HBEC cultures. Tie-bars show paired results from the same donor cultures. 4hr healthy, n = 5; 4hr asthmatic, n = 5; 24hr healthy, n = 6–7; 24hr asthmatic, n = 6. (B) Induction of IL6 and CSF2 by NIST at 4 hours compared to suppression by 100nM 1,25(OH)2D3 at 24 hours of IL-6 and GM-CSF production respectively in NIST PM stimulated HBEC cultures. n = 8 (4 healthy and 4 asthmatic). (TIFF) [file pone.0200040.s004.tiff]

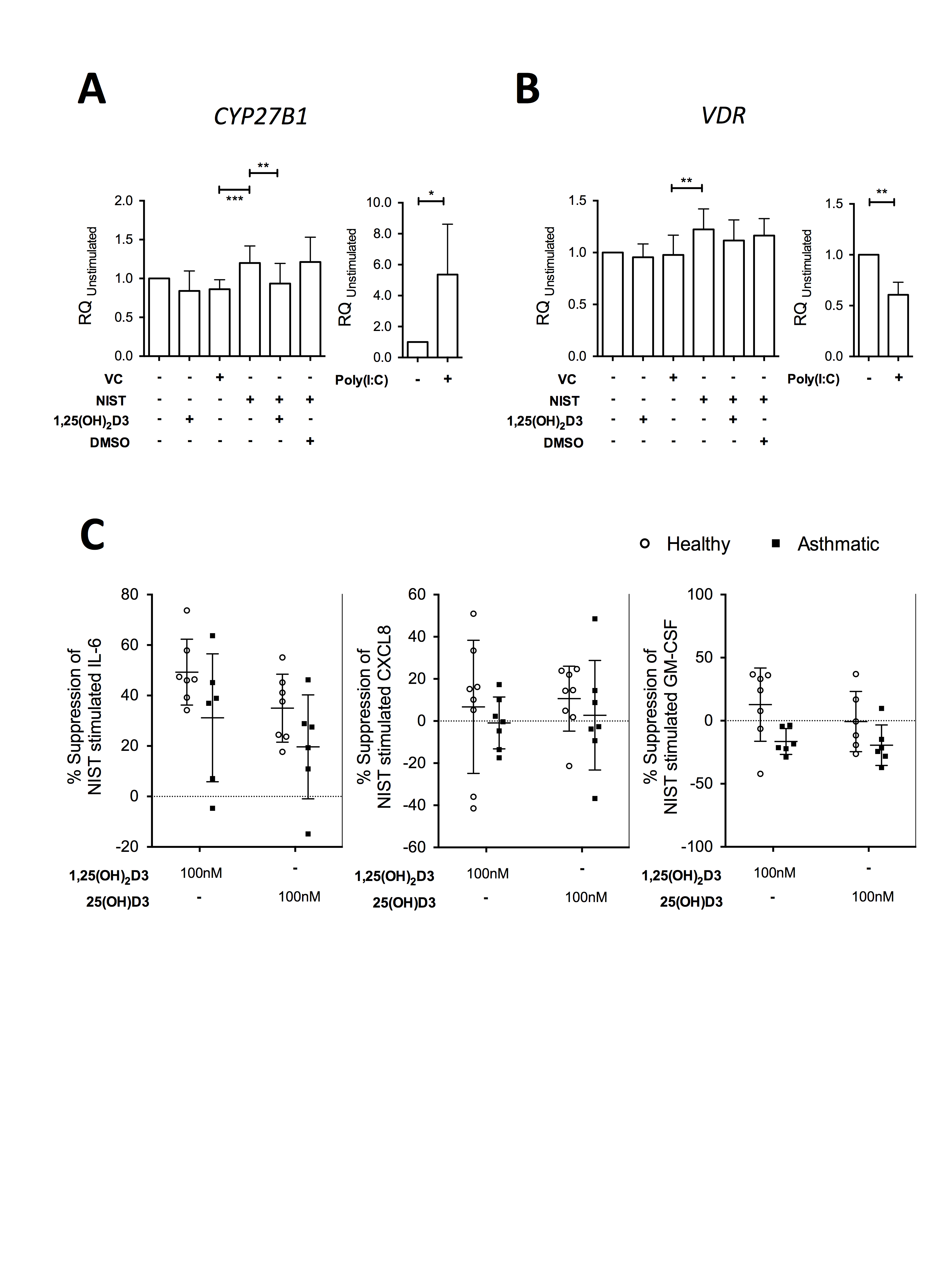

Supplement: S3 Fig — (A) Expression of CYP27B1 mRNA in 24 hour HBEC cultures stimulated with 50μg/ml NIST PM with/without 100nM 1,25(OH)2D3 (n = 9), or with 1μg/ml Poly(I:C) (paired t-test, n = 6). Gene expression measured by qPCR relative to the house-keeping gene 18S, and shown relative to the control condition. Statistical significance as follows: *, p ≤ 0.05; **, p ≤ 0.01; ***, p ≤ 0.001. (B) Expression of VDR in 24 hour cultures stimulated with PM with/without 1,25(OH)2D3 (n = 7), or with 1μg/ml Poly(I:C) (paired t-test, n = 4). (C) Percentage suppression of cytokine-production in 24 hour cultures of PM-stimulated HBECs upon concurrent addition of 1,25(OH)2D3 or 25(OH)D3. Cultures from healthy donors shown as open circles and those from asthmatic donors as filled squares. IL-6, n = 7 healthy, 6 asthmatic; CXCL8, n = 8 healthy, 7 asthmatic; GM-CSF, n = 6–7 healthy, 6 asthmatic. (TIFF) [file pone.0200040.s005.tiff]
